# Prognosis of programmed ventricular stimulation in adult patients with syncope of unexplained origin: A historical cohort

**DOI:** 10.1002/joa3.12953

**Published:** 2023-11-15

**Authors:** Bruno Schaaf Finkler, Roberto Sant'Anna, Javier Pinos, Danilo Barros Zanotta, Thiago Camargo Moreira, Felipe Della Barba de Jesus, Pedro Dutra Batista, Helena Guedes da Rocha, Barbara Adelmann de Lima, Marco Aurélio Lumertz Saffi, Gustavo Glotz de Lima, Marcelo Kruse, Tiago Luiz Luz Leiria

**Affiliations:** ^1^ Instituto de Cardiologia do Rio Grande, do Sul, Fundação Universitária de Cardiologia Porto Alegre RS Brazil; ^2^ Universidade Federal de Ciências da Saúde de Porto Alegre Porto Alegre RS Brazil; ^3^ Hospital de Clínicas de Porto Alegre Porto Alegre RS Brazil

**Keywords:** cohort studies, electrophysiologic study, prognosis, syncope, ventricular tachycardia

## Abstract

**Background:**

Programmed ventricular stimulation (PVS) during electrophysiological study (EPS), is a globally accepted tool for risk stratification of sudden cardiac death (SCD) in some specific clinical situations. The aim of this study was to evaluate the prognosis of ventricular arrhythmia induction in a cohort of patients with syncope of undetermined origin (SUO).

**Methods:**

This is a historical cohort study in a population of patients with SUO referred for EPS between the years 2008–2021. In this interval, 575 patients underwent the procedure.

**Results:**

Patients with induced ventricular arrhythmias had a higher occurrence of structural heart disease (36.7% vs. 76.5%), ischemic heart disease (28.2 vs. 57.1%), heart failure (15.5% vs. 34.4%), and lower left ventricular ejection fraction (59.16% vs. 47.51%), when compared to the outcome with a negative study. PVS triggered ventricular arrhythmias in 98 patients, 62 monomorphic and 36 polymorphic. During a median follow‐up of 37.6 months, 100 deaths occurred. Only the induction of sustained ventricular arrhythmias showed a significant association with the primary outcome (all‐cause mortality) with a *p* value <.001. After the performance of EPS, 142 patients underwent cardioverter‐defibrillator (ICD) implantation. At study follow‐up, 30 patients had therapies by the device. Only the induction of sustained monomorphic ventricular arrhythmia showed statistically significant association with appropriate therapies by the device (*p* = .012).

**Conclusion:**

In patients with SUO, the induction of sustained monomorphic ventricular arrhythmia after programmed ventricular pacing is related to a worse prognosis, with a higher incidence of mortality and appropriate therapies by the ICD.

## Graphical abstract

Induction of sustained monomorphic ventricular arrhythmia during PVS is characterized by higher all‐cause mortality rates and a greater likelihood of receiving appropriate therapies from ICDs. Patients with induced ventricular arrhythmias exhibited a higher prevalence of structural heart disease, ischemic heart disease, and heart failure, along with lower left ventricular ejection fractions when compared to those with negative study outcomes. This study underscores the value of PVS in risk stratification for sudden cardiac death in SUO patients.

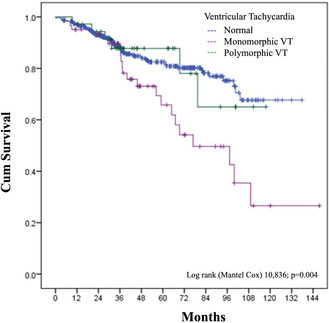

## 1 INTRODUCTION

Syncope, defined as a transitory loss of consciousness and postural tone followed by spontaneous recovery, is a common symptom that corresponds to approximately 3% of the complaints in emergency care and up to 6% of hospital admissions.^1^, ^2^ Even with the use of complementary exams, up to 40% of the cases do not present a clear elucidation for the syncope diagnosis, being this picture classified as syncope of undetermined origin (SUO).^3^ The presence of syncope is associated with increased mortality regardless of its cause, and that it often occurs in patients with underlying heart disease.^4^

Programmed ventricular stimulation (PVS), as part of the electrophysiological study (EPS), is a globally accepted tool for risk stratification of sudden cardiac death (SCD) in patients with structural heart disease and presenting with SUO,^5^ and is even useful in identifying patients at low risk for syncope of arrhythmic origin, when its result is negative in inducing sustained ventricular arrhythmias. However, there is still great uncertainty about the real prognostic value of inducing ventricular arrhythmias in other scenarios, such as patients with preserved ejection fraction, non‐ischemic heart failure, and in the absence of structural heart disease. There is also disagreement about the difference in prognosis in patients with monomorphic ventricular tachycardia (MVT) and polymorphic ventricular tachycardia/ventricular fibrillation (VT/VF) induction.^6^, ^7^, ^8^ The aim of this study was to evaluate the prognosis of ventricular arrhythmia induction in a cohort of patients with SUO.

## 2 METHODS

This is a historical cohort study, which included patients with SUO and who underwent EPS at the *Instituto de Cardiologia do Rio Grande do Sul ‐ Fundação Universitária de Cardiologia (IC/FUC)*. The study was approved by the ethics and research committee of the institution and is in accordance with the guidelines of the Declaration of Helsinki.

### 2.1 Inclusion criteria

All adult patients with a diagnosis of SUO, who underwent EPS in the period from January/2008 to December/2021, were included in the study. Patients' clinical data and test results (laboratory and electrocardiogram), as well as controls after EPS, were recorded in physical and electronic medical records. The EPS data were obtained from the electrophysiology records.

### 2.2 Exclusion criteria

Patients with a diagnosis of syncope suggestive of neurocardiogenic etiology or a clinical condition with a previous ICD indication, and those who could not be contacted to confirm their current clinical status were excluded. We also excluded patients with clinical conditions in which the programmed ventricular stimulation for stratification of sudden death risk is already well defined by the current literature, such as patients with Brugada syndrome, arrhythmogenic right ventricular dysplasia, long QT syndrome, short QT syndrome, and hypertrophic cardiomyopathy (condition in which its performance is not recommended).

### 2.3 Primary outcome

‐All‐cause mortality rate.

### 2.4 Secondary outcomes

‐Incidence of mortality from cardiovascular causes.

‐ICD implantation rate.

‐Appropriate ICD therapies.

‐Syncope recurrence.

### 2.5 Clinical data

Age, sex, diseases such as hypertension, type II diabetes mellitus, coronary artery disease (CAD), congestive heart failure (CHF), and the presence of structural heart disease were collected from the medical history. Left ventricular ejection fraction (LVEF) was obtained from echocardiogram data.

### 2.6 Electrophysiologic study

The procedures were performed in the Electrophysiology Laboratory of our institution with a fluoroscopy device. The data related to the results of the electrophysiological study were evaluated by analyzing the study in the electrophysiology polygraph *(WorkMate™ system—St Jude Medical/Abbot)*. All patients underwent intravenous sedation with propofol (100–150 mcg/kg/min), midazolan (0.02–0.04 mg/kg) and fentanyl (0.5–2 mcg/kg). The right femoral veins were punctured using two introducer sheaths. Fluoroscopy with traditional technology was used as a reference for the introduction and placement of two quadripolar catheters in their specific locations, initially in right atrium and His bundle, and later the right atrium catheter was moved to the right ventricle for the ventricular pacing protocol. The ventricular pacing protocol consists of performing at least two basal cycles of ventricular pacing (600 and 400 ms) with 8 stimuli plus extra stimuli (one, two, or three extra stimuli) at the tip of the right ventricle. All protocols were performed without isoproterenol infusion.

The prematurity of the extra stimulus was limited to a minimum coupling of 200 ms. Those patients who induced nonsustained and sustained VT or nonsustained and sustained VT/VF were considered positive. The criteria for defining sustained ventricular arrhythmia were duration longer than 30 s or association with hemodynamic instability.

### 2.7 Definition of variables

*Mortality*: defined as death from any cause. It was obtained from the Central Civil Registry of the State of *Rio Grande do Sul* and confirmed through calls to the numbers registered in the hospital database.

*Coronary artery disease*: obstruction of the coronary arteries diagnosed by any currently recommended imaging method or have a history of acute coronary syndrome with demonstration of coronary lesions, previous myocardial infarction, or history of coronary angioplasty.

*Congestive heart failure*: defined according to the diagnostic criteria for heart failure, in addition to having had at least one episode of water retention or pulmonary congestion requiring medical treatment.

*Structural heart disease*: any alteration in heart structure and function, demonstrated by echocardiography, CT scan or cardiac magnetic resonance imaging, in the latter including the presence of cardiac fibrosis.

### 2.8 Clinical follow‐up

All information was obtained from the physical or electronic medical records regarding clinical history, physical examination, laboratory tests (including ECG and echocardiogram), procedures, outpatient care, emergency care, and in‐hospital death record. Clinical follow‐up was performed until December 31, 2021. All patients were contacted by telephone in July 2022 to obtain information about the occurrence of death outside the hospital. Patients were excluded from the study if it was not possible to establish any form of contact to determine the patient's status at the end of the follow‐up period. The present study was conducted following the guidelines of the Strengthening the Reporting of Observational Studies (Strobe statement).^9^

### 2.9 Statistical analysis

The database was generated using SPSS Mac OS version 25.0 software *(IBM SPSS Statistics, Chicago, Illinois)*. Continuous variables were described as mean ± standard deviation (SD) or median with 95% confidence interval for that value. Categorical variables were represented as absolute numbers and percentages. A descriptive analysis of clinical, ECG, and EPS‐related variables was performed. All‐cause death‐free survival was assessed by the Kaplan–Meier method. The significance level was set as *p* < .05. Univariate analysis was performed by the *χ* ^2^ test, Fisher's exact test, analysis of variance, or the t‐test, as necessary. Those variables with *p* < .05 were included for multivariate analysis. Independent predictors of all‐cause death were analyzed by multivariable logistic regression.

## 3 RESULTS

**FIGURE 1:**
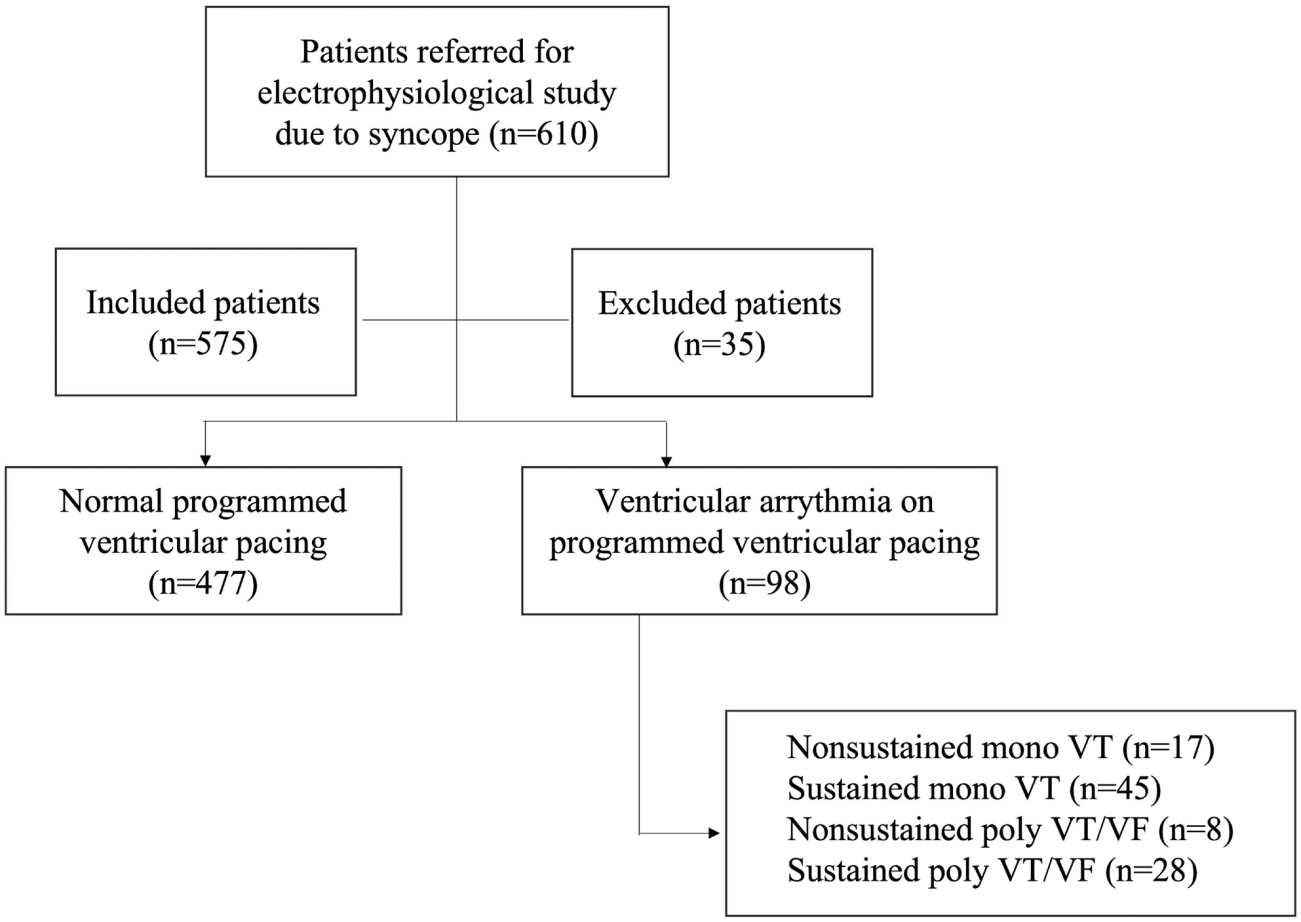
Study flowchart. VF, ventricular fibrillation; VT, ventricular tachycardia.

Between the years 2008–2021, 610 patients were referred to perform an EPS due to syncope of undetermined origin in the institution. Of these, 35 were excluded because they did not meet the inclusion criteria of the study (15 patients without a clinical picture suggestive of syncope, 9 patients with Brugada Syndrome, 6 patients with suspected arrhythmogenic right ventricular dysplasia, 5 patients with hypertrophic cardiomyopathy). Thus, 575 patients were included in the final analysis of the study (Figure 1). Of the patients evaluated, 56.1% had only 1 episode of syncope and 25.4% were hospitalized for investigation of the condition. The median follow‐up of patients in this time interval was 37.6 months.

Table 1 shows the characteristics of the study population. The mean age of the patients was 64.5 ± 16.2 years among the patients who did not induce arrhythmias and 66.2 ± 16.3 years among those who induced some type of ventricular arrhythmia. The patients who induced ventricular arrhythmias were mostly male (269–56.8% vs. 80–80.6% ‐ *p* < .001), 72.4% were hypertensive and 27.6% were diagnosed with diabetes mellitus. The diagnosis of heart failure (HF) was present in 33 patients with induction of ventricular arrhythmias (*p* < .001), with 19 (19.8%) presenting heart failure with reduced ejection fraction (HFrEF) of ischemic etiology, 14 (14.6%) nonischemic HFrEF and 6 (18.1%) of valvular etiology. Regarding the previous diagnosis of arrhythmia, 77% of patients had no previous diagnosis, 11.4% had atrial fibrillation, 5.1% had other supraventricular tachyarrhythmias, and around 2% had ventricular ectopy. The measurement of ejection fraction showed a significant difference between patients who did not induce and those who induced ventricular arrhythmias in the EPS (59.16% vs. 47.51%, *p* < .001).

**TABLE 1:** Study population characteristics.

Characteristics	Without induction VT/FV (*n* = 477)	With induction VT/FV (*n* = 98)	*p* value
Age	64.5 ± 16.2	66.2 ± 16.3	.45
Male sex	269 (56.8%)	80 (80.6%)	.001
Multiple episodes of syncope	214 (45.1%)	37 (37.8%)	.185
Hospitalization	104 (21.8%)	42 (42.9%)	.001
Hypertension	314 (66.1%)	71 (72.4%)	.223
Diabetes mellitus	90 (18.95)	27 (27.6%)	.054
Ejection fraction	59.16 ± 13.8%	47.51 ± 14.84%	.001
Structural cardiopathy	172 (36.7%)	75 (76.5%)	.001
Ischemic cardiopathy	133 (28.2%)	56 (57.1%)	.001
Heart faillure (HF)^a^	73 (15.5%)	33 (34.4%)	.001
Ischemic HRrEF	20 (4.3%)	19 (19.8%)	NS
Non‐ischemic HRrEF	31 (6.6%)	14 (14.6%)	NS

^a^ Patients with a clinical diagnosis of heart failure; HFrEF—Heart failure with reduced ejection fraction (<40%); NS—statistically non‐significant “*p*” value.

Performing EVP caused ventricular arrhythmias in 98 patients, Table **2** describes these results. Of these 98 patients, 62 were monomorphic ventricular arrhythmias (17 nonsustained and 45 sustained) and 36 were polymorphic (8 nonsustained and 28 sustained). Regarding mortality, the main outcome of the study, 100 (17.6%) patients died during clinical follow‐up. Among the patients who induced VT, 22 (36%) evolved to death, presenting a statistically significant association in relation to the patients who did not induce arrhythmias [*n* = 22 (35.4%) vs. *n* = 72 (15%); *p* < .001]. The induction of polymorphic ventricular tachycardia showed no significant correlation with the occurrence of death.

**TABLE 2:** Mortality results.

Programmed ventricular stimulation	Deaths (*N* = 100)	No death (*N* = 474)	*p* value
Normal (*n* = 477)	72 (15.1%)	404 (84.9%)	NS*
Non‐sustained monomorphic VT (*n* = 17)	4 (23.5%)	13 (76.5%)	NS*
Sustained monomorphic VT (*n* = 45)	18 (40%)	27 (60%)	*p* < .001
Non‐sustained polymorphic VT/VF (*n* = 8)	2 (25%)	6 (75%)	NS*
Sustained polymorphic VT/VF (*n* = 28)	4 (14.3%)	24 (85.7%)	NS*

Abbreviations: *NS, statistically non‐significant “*p*” value; VT, ventricular tachycardia; VF, ventricular fibrillation.

Clinical follow‐up of the patients showed that 142 patients underwent ICD, and 30 of these had device therapies. Regarding the induction of ventricular arrhythmias in the EPS, only the occurrence of sustained VT was associated with appropriate ICD therapies. Among the 18 appropriate therapies that occurred in the follow‐up, 10 occurred in patients with monomorphic VT induction (27%—*p* = .012). Among patients who underwent ICD implantation even in the absence of induction of ventricular arrhythmias in the EPS (74 patients), only 3 patients had appropriate therapies at clinical follow‐up (Table 3).

**TABLE 3:** Patients undergoing ICD implantation with appropriate therapies.

Programmed ventricular stimulation	ICD—No shock (*n* = 119)	ICD—appropriate therapies (*n* = 18)	ICD—inappropriate therapies (*n* = 12)	*p* value
Normal (*n* = 73)	66 (90.4%)	3 (4.1%)	4 (5.5%)	NS*
Non‐sustained monomorphic VT (*n* = 11)	8 (72.7%)	1 (9%)	2 (18.2%)	NS
Sustained monomorphic VT (*n* = 37)	24 (64.9%)	10 (27%)	3 (8.1%)	.012
Non‐sustained polymorphic VT/VF (*n* = 4)	3 (75%)	1 (25%)	—	NS
Sustained polymorphic VT/VF (*n* = 24)	18 (75%)	3 (12.5%)	3 (12.5%)	NS

Abbreviations: ICD, implantable cardioverter‐defibrillator; *NS, statistically non‐significant “*p*” value; VT, ventricular tachycardia; VF, ventricular fibrillation.

The presence of structural heart disease was a risk factor for the occurrence of ventricular arrhythmias, and 30.4% of them presented triggering of arrhythmias in EPS (*p* < .001). The stratification according to the ventricular ejection fraction showed that the cutoff point below 40% correlated positively with the occurrence of ventricular arrhythmias (*p* < .001).

Regarding the number of ventricular extra stimuli, only the performance of 3 extra stimuli showed a statistically significant relationship with the occurrence of ventricular arrhythmias (*p* < .001).

**FIGURE 2:**
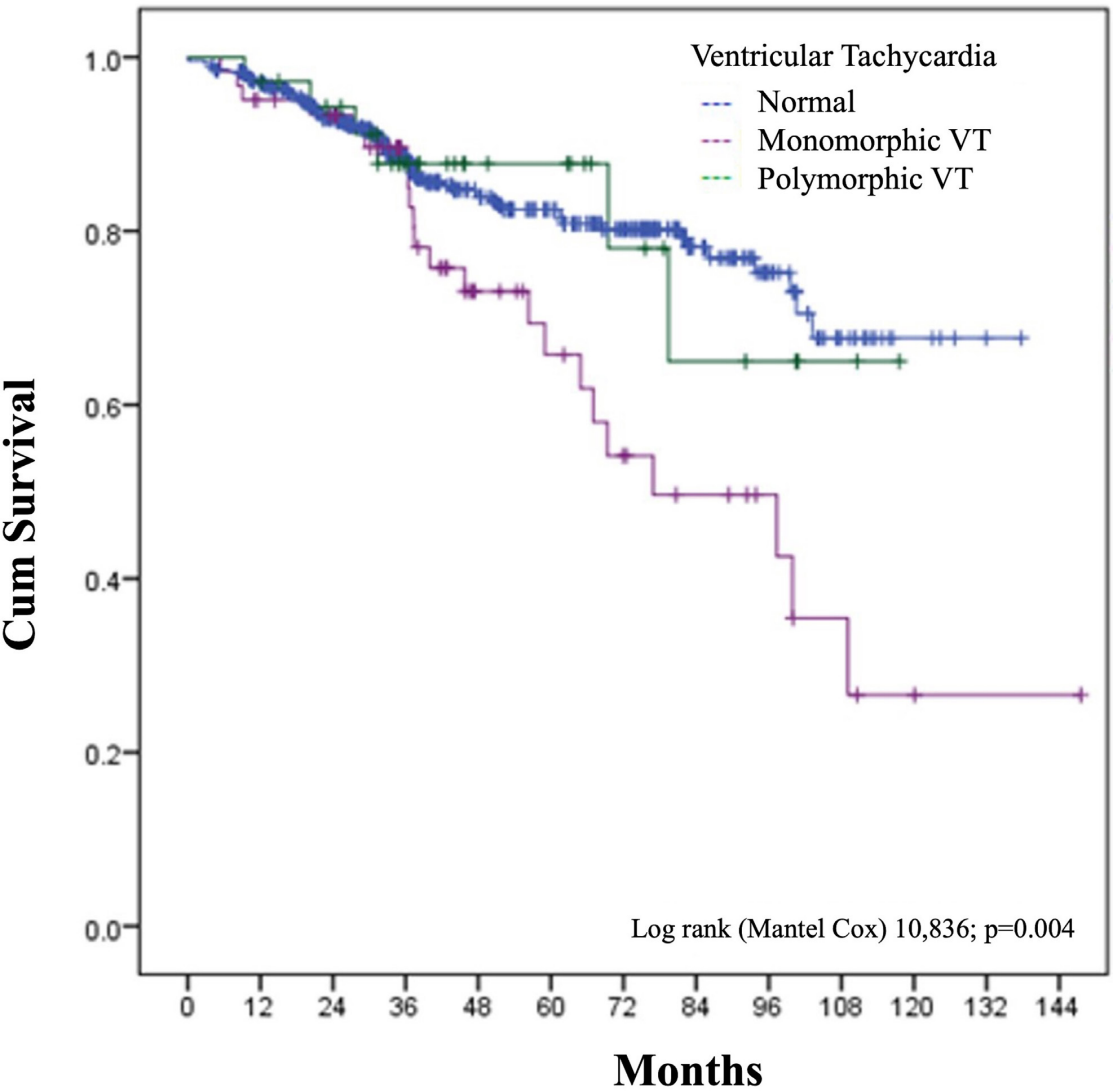
Survival curve. VT, ventricular tachycardia.

The Kaplan curve of survival over time showed that only the induction of monomorphic ventricular arrhythmias was significantly associated with the main outcome (*p* = .004 – log‐rank test) (Figure 2). Stratification with respect to ventricular arrhythmia episodes showed that only the induction of sustained episodes of monomorphic ventricular arrhythmias was significantly associated with mortality (*p* < .001).

Logistic regression was performed using a model in which variables were included using the backward method based on probability. The criteria for entry into the regression analysis were a *p*‐value of .05 and a removal value of 0.20. The variables selected to enter the model were those that showed statistical significance in the univariate analysis and were deemed relevant by the investigators. The logistic regression analysis showed that the independent variables related to mortality were age, heart failure, atrial fibrillation, and induction of monomorphic ventricular arrhythmia (Table 4).

**TABLE 4:** Logistic regression model.

	Death (*n* = 100)	No death (*n* = 474)	OR	CI 95%	*p*
Age (mean, SD)	71.6 ± 11.3	62.6 ± 16.7	1.054	1.0–1.0	<.001
Heart failure	33 (33%)	73 (15.6%)	2.085	1.2–3.6	.009
Basal rhythm AF	13 (13.5%)	22 (4.7%)	3.238	1.3–7.7	.008
Mono‐VT inducible	22 (22%)	39 (8.2%)	3.117	1.6–6.0	.001
Poli‐VT inducible	6 (6%)	30 (6.3%)	1.186	0.4–3.0	.727
HV >70 ms	29 (29.9%)	74 (15.8%)	1.512	0.8–2.7	.164

*Note*: Logistic regression backward conditional model. Variables entered: Age, Basal EKG rhythm, Monomorphic Ventricular Tachycardia (Mono‐VT), Polymorphic Ventricular Tachycardia (Poly‐VT), His‐ventricle interval (HV).

## 4 DISCUSSION

The performance of EPS with PVS is often performed to unmask the cause of syncope in patients who present without a defined etiology for it. The results of the study corroborate some current data in the literature, showing that patients with induction of sustained monomorphic ventricular arrhythmias in EPS of patients with SUO have a higher risk of adverse outcomes than other patients. The induction of sustained ventricular arrhythmias, in addition to being associated with mortality, also showed a positive association with the occurrence of appropriate ICD therapies, identifying patients at high risk for future arrhythmic events. Similarly, *Andrews NP* et al.^10^ published a retrospective case–control study with patients with syncope of undetermined origin, structural heart disease, and spontaneous or induced ventricular arrhythmias on electrophysiological study. In this study, they were able to identify that the incidence of ventricular tachycardia at follow‐up was identical between patients in the control group (patients with VT induced on electrophysiological study) and patients with previously documented spontaneous VT. They also demonstrated the positive correlation between induction of ventricular arrhythmias with the PVS and the occurrence of ICD therapies.

Similarly, *Menon Venum* et al.^11^ demonstrated through a retrospective clinical study with 36 patients with SUO, structural heart disease and ventricular tachycardia induced in EPS, that the rate of ICD therapies was 56% in 36 months. It is noteworthy that the patients in this cohort had a lower EF (mean 31 ± 12%) and more severe ischemic heart disease (one third of patients were surgically revascularized). *Pires LA and colleagues* ^12^ compared survival rates and ICD therapies in 178 patients with syncope of undetermined origin and with induced and ventricular arrhythmias in the EPS (VT or VF) versus 568 patients with syncope and documented ventricular arrhythmias. After a mean follow‐up of 11 ± 10 months, they found that patients with induction of ventricular arrhythmias in the EPS had a similar incidence of death, syncope recurrence, and ICD therapy as patients with documented ventricular arrhythmias.

In the study described above, induction of ventricular fibrillation is suggested to be a less specific finding for future ICD therapies (35% of patients with monomorphic VT had ICD therapies versus 18% with VF induction).

The 1997 *AVID study* ^13^ (Antiarrhythmics Versus Implantable Defibrillators) was one of the major studies to demonstrate the benefit of ICDs for secondary prevention of sudden death. In 2001 *Steinberg, J and colleagues* ^14^ published a sub‐study from the AVID data, including 80 patients with syncope of undetermined origin and with induction of ventricular arrhythmias on EPS: 21 patients (26%) with polymorphic VT/VF, 11 (14%) monomorphic VT with frequency <200 bpm and 48 (60%) monomorphic VT with frequency >200 bpm. In this study it was concluded that induction of ventricular arrhythmias is associated with a higher mortality rate comparable to patients in the AVID study (with spontaneous VT/VF) and future ventricular arrhythmic events. The type of ventricular arrhythmia induced did not show a significant difference in the outcomes studied.

The data demonstrated in the present study, showed that the induction of sustained monomorphic ventricular arrhythmias showed an association with mortality regardless of the presence or absence of associated structural heart disease, although the presence of structural heart disease and reduced EF were independent factors for the occurrence of VT. Seeking to evaluate risk stratification in patients with syncope and ischemic heart disease or left ventricular dysfunction, *Bembrila‐Perrot, B* et al.,^15^ demonstrated in their study that the induction of VT in patients with ischemic heart disease was significantly associated with the occurrence of sudden death (*p* < .01), with respect to patients in whom it was not possible to induce ventricular arrhythmias. The study shows that the positive predictive value of inducing ventricular arrhythmias is low (18%) for predicting sudden death, but with high negative predictive value (97%) for the same outcome.

To verify the effect of programmed ventricular pacing in patients with SUO and idiopathic ventricular dysfunction, *Brilaskis ES and colleagues* ^16^ performed a systematic review of the literature. Through the review, the authors conclude that PVS was able to induce sustained ventricular arrhythmias in 53% of the cases and is associated with a higher rate of appropriate ICD therapies. However, they also demonstrated that in a 28‐month follow‐up, 24% of the patients with negative results in ventricular pacing presented appropriate shocks by ICD. These data suggest that ventricular pacing with a negative result may not have as favorable a prognosis in patients with dilated cardiomyopathy as in those with ischemic heart disease.

The morphology of the induced ventricular arrhythmia in the electrophysiological study seems to be related to the prognosis of new future arrhythmic events, ICD therapies, and mortality. Similar findings were demonstrated by *Mittel S* et al.^17^ who presented that in patients with ischemic heart disease and syncope of undetermined origin, the induction of VF with programmed pacing showed no difference in patient survival during follow‐up, demonstrating a low specificity of the induction of this ventricular arrhythmia morphology, especially when using more aggressive pacing protocols, with three extrastimuli.

*Gurbhej Singh and colleagues* ^18^ conducted a retrospective study analyzing the induction of ventricular arrhythmias in patients with structural heart disease. They showed that the induction of ventricular arrhythmias was associated with the occurrence of ischemic heart disease and left ventricular dysfunction. Similarly, to the present study, they identified that the occurrence of sustained monomorphic ventricular tachycardia was associated with ICD shocks (*p* < .001) and VT recurrence (*p* < .001) in the follow‐up, but with no association with mortality (*p* = .139).

The PVS has an important role in risk stratification in patients with syncope of undetermined origin. The present study, despite its retrospective design, presents a long follow‐up of patients with syncope without defined etiology who underwent EPS as an auxiliary diagnostic method, showing that patients with monomorphic ventricular arrhythmias have a higher rate of mortality and appropriate therapies by ICD, without stratification by the presence or absence of baseline structural heart disease.

The most current guidelines on syncope and sudden death prevention support the use of electrophysiologic study with programmed ventricular pacing for risk stratification of patients with syncope. The guideline of the American Heart Association classifies as recommendation class IIa, level of evidence B, the performance of EPS with ventricular pacing for patients with syncope and ischemic heart disease, nonischemic dilated heart disease or adults with congenital heart disease who do not meet criteria for ICD as primary prevention.^18^ The European Society of Cardiology guideline describes as a strong prognostic factor the induction of sustained monomorphic ventricular arrhythmias in patients with previous myocardial infarction, whereas the induction of ventricular fibrillation is considered a nonspecific finding. They classify as recommendation class I, level of evidence B, the performance of EPS in patients with syncope and previous infarction, with inconclusive non‐invasive investigation, and recommendation class IIb, level of evidence C, for patients with syncope preceded by palpitations. They further describe that induction of polymorphic VT and VF in patients with nonischemic dilated cardiomyopathy and ischemic heart disease should not be interpreted as causes of syncope.^19^, ^20^

### 4.1 Limitations

This work presents limitations, starting with its retrospective design, which to a certain extent limits the extrapolation of its results. The ventricular pacing protocol performed was the responsibility of the electrophysiologist responsible for the procedure, with no predetermined standard for each specific clinical situation, which can limit its evaluation, but at the same time reflects a situation of real clinical practice, considering that, as has been reviewed in the current guidelines, there is no description of specific protocols to be followed in this clinical situation. We also highlight as a limiting factor the small number of patients in each subgroup of patients with induction of ventricular arrhythmias, after stratification by morphology and duration.

## 5 CONCLUSION

The present study is a large cohort of patients with syncope of undetermined origin undergoing electrophysiological study, showing that the induction of sustained monomorphic ventricular arrhythmia, after programmed ventricular pacing, is related to a worse prognosis, with a higher incidence of mortality and appropriate ICD therapies, regardless of stratification by the presence of structural heart disease.

## CONFLICT OF INTEREST STATEMENT

There are no conflicts of interest or financial disclosures to declare regarding this study.

## ETHICS APPROVAL STATEMENT

This study was approved by the research ethics committee under number 5256/16.

## PATIENT CONSENT STATEMENT

Written informed consent for the ablation and participation in this study was obtained from all patients.

## CLINICAL TRIAL REGISTRATION

N/A.

## References

[1] Grossman SA , Badireddy M . Syncope. [Updated 2022 Jun 21]. In: StatPearls. Treasure Island (FL): StatPearls Publishing; 2022.

[2] Manolis AS , Linzer M , Salem D , Estes NAM III . Syncope: current diagnostic evaluation and management. Ann Intern Med. 1990;112:850–863. 2188544 10.7326/0003-4819-112-11-850

[3] Kapoor WN . Evaluation and outcome of patients with syncope. Med. 1990;69:160–175. 10.1097/00005792-199005000-00004 2189056

[4] Link MS , Kim KM , Homoud MK , Estes NA III , Wang PJ . Long‐term outcome of patients with syncope associated with coronary artery disease and a nondiagnostic electrophysiologic evaluation. Am J Cardiol. 1999;83:1334–1337. 10235091 10.1016/s0002-9149(99)00096-x

[5] Shen WK , Sheldon RS , Benditt DG , Cohen MI , Forman DE , Goldberger ZD , et al. 2017 ACC/AHA/HRS guideline for the evaluation and management of patients with syncope: a report of the American College of Cardiology/American Heart Association task force on clinical practice guidelines and the Heart Rhythm Society. Circulation. 2017;136(5):e60–e122. 28280231 10.1161/CIR.0000000000000499

[6] Kushner JA , Kou WH , Kadish AH , Morady F . Natural history of patients with unexplained syncope and a nondiagnostic electrophysiologic study. J Am Coll Cardiol. 1989;14:391–396. 2754128 10.1016/0735-1097(89)90191-5

[7] Klein GJ , Gersh BJ , Yee R . Electrophysiological testing: the final court of appeal for diagnosis of syncope? Circulation. 1995;92:1332–1335. 7648683 10.1161/01.cir.92.5.1332

[8] Brugada P , Green M , Abdollah H , Wellens HJ . Significance of ventricular arrhythmias initiated by programmed ventricular stimulation: the importance of the type of ventricular arrhythmia induced and the number of premature stimuli required. Circulation. 1984;69:87–92. 6689650 10.1161/01.cir.69.1.87

[9] Von Elm E , Altman DG , Egger M , Pocock SJ , Gotzsche PC , Vandenbroucke JP , et al. The strengthening the reporting of observational studies in epidemiology (STROBE)statement: guidelines for reporting observational studies. Lancet. 2007;370(9596):1453–1457. 18064739 10.1016/S0140-6736(07)61602-X

[10] Andrews NP , Fogel RI , Pelargonio G , Evans JJ , Prystowsky EN . Implantable desfibrillator event rates in patients with unexplained syncope and inducible sustained ventricular tachyarrhythmias. JACC. 1999;34(7):2023–2030. 10588219 10.1016/s0735-1097(99)00465-9

[11] Menon V , Steinberg JS , Akiyama T , Beckman K , Carillo L , Kutalek S . ICD discharge rates in structural heart disease. Clin Cardiol. 2000;23:195–200. 10761808 10.1002/clc.4960230312 PMC6655031

[12] Pires LA , May LM , Ravi S , Parry JT , Lal VR , Nino CL . Comparison of event rates and survival in patients with unexplained syncope without documented ventricular tachyarrhythmias versus patients with documented sustained ventricular tachyarrhythmias both treated with implantable cardioverter‐defibrillators. Am J Cardiol. 2000;85:725–728. 12000047 10.1016/s0002-9149(99)00848-6

[13] Zipes DP , Wyse DG , Friedman PL , Epstein AE , Hallstrom AP , Greene HL , et al. A comparison of antiarrhythmic‐drug therapy with implantable defibrilattor in patients resuscitaded from near‐fatal ventricular arrhythmias the antiarrhythmics versus implantable defibrillatiors (AVID). N Engl J Med. 1997;337(22):1576–1583. 9411221 10.1056/NEJM199711273372202

[14] Nathan S , Steinberg MD . Follow‐up of patients with unexplained syncope and inducible ventricular tachyarrhythmias: analysis of the AVID registry and an AVID substudy. J Cardiovasc Electrophysiol. 2001;12(9):996–1001. 11573709 10.1046/j.1540-8167.2001.00996.x

[15] Be'atrice Brembilla‐Perrot, MD . Differences in mechanisms and outcomes of syncope in patients with coronary disease or idiopathic left ventricular dysfunction as assessed by electrophysiologic testing. J Am Coll Cardiol. 2004;44(3):594–601. 15358027 10.1016/j.jacc.2004.03.075

[16] Brilakis ES , Emmanouil S , Brilakis A , Friedmana PA , Maounisb TN , Rokasc SG , et al. Cokkinosb programmed ventricular stimulation in patients with idiopathic dilated cardiomyopathy and syncope receiving implantable cardioverter‐defibrillators: a case series and a systematic review of the literature. Int J Cardiol. 2005;98:395–401. 15708170 10.1016/j.ijcard.2003.12.012

[17] Mittal S , Hao SC , Iwai S , Stein KM , Markowitz SM , Slotwiner DJ , et al. Significance of inducible ventricular fibrillation in patients with coronary artery disease and unexplained syncope. J Am Coll Cardiol. 2001;38(2):371–376. 11499726 10.1016/s0735-1097(01)01379-1

[18] Singh G , Prasad S , Namboodiri N , Thajudeen A , Nair KKM , Abhilash SP , et al. Programmed ventricular stimulation in structural heart disease: implications of patterns of ventricular arrhythmias induced to long term outcomes. Indian Heart J. 2023;75:17–24. 36581158 10.1016/j.ihj.2022.12.002 PMC9986739

[19] Al‐Khatib SM , Stevenson WG , Ackerman MJ , Bryant WJ , Callans DJ , Curtis AB , et al. AHA/ACC/HRS guideline for management of patients with Ventricular Arrhythmias and the prevention of sudden cardiac death a report of the American College of Cardiology/American Heart Association task force on clinical practice guidelines and the Heart Rhythm Society September 25, 2018. Circulation. 2017;138:e272–e391. 10.1161/CIR.0000000000000549 29084731

[20] Brignole M , Moya A , Lange FJ , Deharo JC , Elliot PM , Fanciulli A , et al. 2018 ESC guidelines for the diagnosis and management of syncope the task force for the diagnosis and management of syncope of the European Society of Cardiology (ESC) authors/task force members: Michele Brignole* (chairperson). Eur Heart J. 2018;39:1883–1948. 29562304 10.1093/eurheartj/ehy037

